# Treatment effect analysis of the Frailty Care Bundle (FCB) in a cohort of patients in acute care settings

**DOI:** 10.1007/s40520-024-02840-5

**Published:** 2024-09-10

**Authors:** Colum Crowe, Corina Naughton, Marguerite de Foubert, Helen Cummins, Ruth McCullagh, Dawn A. Skelton, Darren Dahly, Brendan Palmer, Brendan O’Flynn, Salvatore Tedesco

**Affiliations:** 1 Tyndall National Institute, University College Cork, Lee Maltings Complex, Dyke Parad, T12R5CP Cork, Ireland; 2 School of Nursing and Midwifery, University College Cork, Cork, T12 AK54 Ireland; 3 School of Clinical Therapies, University College Cork, Cork, T12 AK54 Ireland; 4 School of Health and Life Sciences, Glasgow Caledonian University, Glasgow, G4 0BA Scotland, UK; 5 HRB Clinical Research Facility-UCC, and School of Public Health, University College Cork, Cork, T12 XF62 Ireland

**Keywords:** Nursing, Older people, Mobilization, Hospital Associated decline, Functional decline, Accelerometry, Machine learning

## Abstract

**Purpose:**

The aim of this study is to explore the feasibility of using machine learning approaches to objectively differentiate the mobilization patterns, measured via accelerometer sensors, of patients pre- and post-intervention.

**Methods:**

The intervention tested the implementation of a Frailty Care Bundle to improve mobilization, nutrition and cognition in older orthopedic patients. The study recruited 120 participants, a sub-group analysis was undertaken on 113 patients with accelerometer data (57 pre-intervention and 56 post-intervention), the median age was 78 years and the majority were female. Physical activity data from an ankle-worn accelerometer (StepWatch 4) was collected for each patient during their hospital stay. These data contained daily aggregated gait variables. Data preprocessing included the standardization of step counts and feature computation. Subsequently, a binary classification model was trained. A systematic hyperparameter optimization approach was applied, and feature selection was performed. Two classifier models, logistic regression and Random Forest, were investigated and Shapley values were used to explain model predictions.

**Results:**

The Random Forest classifier demonstrated an average balanced accuracy of 82.3% (± 1.7%) during training and 74.7% (± 8.2%) for the test set. In comparison, the logistic regression classifier achieved a training accuracy of 79.7% (± 1.9%) and a test accuracy of 77.6% (± 5.5%). The logistic regression model demonstrated less overfitting compared to the Random Forest model and better performance on the hold-out test set. Stride length was consistently chosen as a key feature in all iterations for both models, along with features related to stride velocity, gait speed, and Lyapunov exponent, indicating their significance in the classification.

**Conclusion:**

The best performing classifier was able to distinguish between patients pre- and post-intervention with greater than 75% accuracy. The intervention showed a correlation with higher gait speed and reduced stride length. However, the question of whether these alterations are part of an adaptive process that leads to improved outcomes over time remains.

**Supplementary Information:**

The online version contains supplementary material available at 10.1007/s40520-024-02840-5.

## Introduction

Older adults represent one of the most vulnerable groups of patients in acute care hospitals [1]. The hospitalization process can lead to the development of functional or cognitive impairments, referred to as hospital associated decline (HAD). Older patients admitted to hospitals today exhibit distinct heath profiles in comparison to the past. Due to the changes in population demographics in the last few decades, an increasing number are presenting with more co-morbidities including physical and cognitive frailty, and polypharmacy [2]. In addition, acute care health systems, still mainly operate a single disease model that are poorly adapted to meet the more complex needs of this growing patient population [3]. Thus, older adults are at a higher risk of HAD and other adverse events leading to extended hospital stays [4].

HAD refers to the development of new functional or cognitive deficits during hospitalization that were not present upon admission [5]. The main effects of HAD include reduced mobility and loss of independence in daily activities [6]. The prevalence of HAD in older hospitalized patients ranges from 33 to 40%, and it can persist for several months after discharge [6]. Several factors contribute to HAD, including the adoption of traditional disease-focused care models, a lack of emphasis on fundamental care, and inadequate attention to early mobilization [5]. Adverse outcomes related to HAD include functional decline, falls, delirium, pressure ulcers, delayed discharge, re-admissions, and transitioning to institutionalized care [5].

Efforts to address HAD include ward-based interventions that aim to prioritize fundamental care activities such as early and consistent mobilization, optimizing nutrition intake (particularly protein), and preserving cognitive function through cognitive orientation and stimulation. However, very few interventions have taken all three aspects into consideration [7–9].

As part of the efforts to tackle HAD, the Frailty Care Bundle (FCB) project developed an innovative intervention combining evidence-based approaches for early mobilization, improved nutrition, and enhanced cognitive engagement; with the overall goal of creating a comprehensive care package to prevent the decline of older patients’ physical function during their hospital stay [10]. The study intervention and outcomes are more fully described elsewhere [11], but briefly, despite COVID-19 restrictions, the FCB demonstrated a non-significant increase in the probability of patients returning to their pre-injury functional capability, measured using the modified Barthel Index (mBI), in the post-intervention group (odds ratio 2.29, 95% confidence intervals 0.98–5.63) compared to those in the pre-intervention group. One of the primary study objectives was to increase patients’ mobilization during hospitalization, which was measured using off-the-shelf accelerometers. This data indicated an average 11% increase in-hospital patient step count in the FCB post-intervention group [11].

The adoption of wearable sensors in estimating physical activity in patients in acute care settings is not a new development. For instance, several studies adopted accelerometers to evaluate independent physical activity (in the majority of cases, in terms of step counts or time spent walking) in hospitalized subjects following cardiac surgery [12, 13], in intensive care units [14], or rehabilitation wards [11, 15–17]. However, those studies did not consider machine learning as a tool for discriminating between pre- and post-intervention patients. Machine learning is currently widely employed as a tool for the recognition of hidden patterns in data, with a substantial body of research focusing on its application in healthcare datasets involving wearable accelerometry data [18–20]. Among the referenced studies [12–15], only Halfwerk et al. [12] implemented an artificial neural network in their work. However, their focus was on classifying various patient activities (e.g., lying in bed, sitting in a chair, standing, walking, cycling on an exercise bike, and walking the stairs) rather than assessing the potential impact and effects of an intervention on patient outcomes.

The primary aim of this present study is to explore the feasibility of using machine learning approaches to objectively differentiate the mobilization patterns, measured via accelerometer sensors, of patients pre- and post-intervention. This investigation seeks to more fully understand the potential positive impacts of the intervention on patients’ mobilization as an intermediary step in preserving or regaining patients’ functional capability as measured by the modified Barthel Index. To the best of the authors’ knowledge, this is the first application of machine learning to accelerometry data collected in the context of HAD.

## Methods

### Frailty Care Bundle (FCB) intervention

The Frailty Care Bundle (FCB) intervention aimed to reduce risk of HAD by implementing evidence-based practices to promote mobilization, nutrition and cognitive engagement [11]. The FCB intervention study was undertaken with patients requiring surgical intervention following orthopaedic trauma and who were managed on two acute care surgical wards and two orthopaedic rehabilitation wards during their recovery. The study employed a two-group pre-post intervention design.

The FCB principles were tailored to each ward based on a detailed ward situational analysis and input from the nursing, multidisciplinary teams (MDT) and local implementation group. The FCB principles included [11]:

### Early & consistent mobilization

- Mobilization assessment by physiotherapist within 24 h of surgery or transfer to rehabilitation.
- Individualised Patient Mobility goal setting (aim for minimum of three times a day aligned to baseline function).
- Provision of mobilization assistance (as appropriate) and supervision by nursing team (in addition to scheduled physiotherapy).

### Enhanced nutrition

- Increase supervision and assistance at mealtimes.
- Reduce disruption at mealtimes.
- Nutrition screening and weekly re-appraisal.
- Increase availability of high protein and calorie food (e.g. enhanced drinks round and protein snacks).

### Cognitive engagement

- Increase cognitive engagement activities among patients (talking, on-line games, reading, listening to radio).
- Improvement in environment layout to promote orientation and patient mobilization.

The study received ethical approval from the Clinical Research Ethics Committee of the Cork Teaching Hospitals (Ref ECM 4 (d) 10/09/2019) and the study protocol was registered with ISRCTN 10.1186/ISRCTN15145850). The FCB intervention was regarded as best clinical practice and thus all patients were eligible to receive the intervention without the need for written consent; however, all participants recruited as part of the evaluation of the FCB provided written informed consent for data collection to measure the effect of the intervention on patient outcomes.

The therapeutic goal of orthopaedic trauma care is to restore patient mobilization and baseline functional capability, thus the priority for change was to increase patients’ opportunity to mobilize through the assistance of the nursing team, in addition to patients’ scheduled physiotherapy [11]. Across the four wards, observation data at baseline, identified low levels of patient mobilization and prolonged sedentary periods during the day, for example on average, 83% of patients were sitting out of bed (minimum 71% to maximum 93%), the median time spent walking was 8% (min 5%, max 16%) compared to time sitting in a chair was 44% (min 43%, max 62%) [11]. Observation data on nutrition intake, suggested that in 56% (min 45%, max 68%) of meals provided, less than half the meal was eaten, while 27% of patients (15–34%) were identified as cognitively vulnerable (confused on admission) or had a diagnosis of dementia.

The intervention entailed a clinical facilitator working with clinical teams and using plan-do-study-act (PDSA) cycles, to implement strategies to increase patient mobilization. Strategies included patient mobility goal setting and improved information exchange between nursing, physiotherapy, and medical teams. Using either an individual patient mobilization sheet or mobility (white) board, patients’ mobility capability (level of assistance and equipment required) and daily mobility goal (e.g. walk 10 m, three times a day) was made visible to patients and the bedside nurse with the expectation that nurse assisted mobilization would increase [11]. To determine the efficacy of this approach in improving outcomes for older patients and to gain a deeper understanding of its mechanisms, it is necessary to assess it through the analysis of objective measures.

Daily step counts and other gait parameters were collected, using a shank-worn off-the-shelf accelerometer (StepWatch 4; https://modushealth.com/), to be used as a proxy measure for patient functional capability and in-hospital mobilization.

The intervention also involved nutrition changes to increase the opportunities for food intake through the provision of enhanced snack/hydration rounds to include protein snacks combined with re-emphasising assisted and protected mealtime principles. Cognitive engagement changes were introduced on the rehabilitation site including the provision of patient activity packs, and environmental changes to improve orientation, while delirium screening was a priority on the acute care wards [11]. In this paper, we are interested in the patient mobilization aspect of the intervention, the details on nutrition and cognition are reported elsewhere [11].

### Patient selection

The initial sample size target was *n* = 180, consisting of 90 patients in the pre-intervention group and 90 patients in the post-intervention group, giving 80% power to detect a significant difference [10, 11]. The original intention was to include medical and surgical population, but due to disruption from COVID-19, it was not possible to complete the project on the medical wards. In this paper we have focused on the surgical population only. The intervention was delivered across two hospitals and four wards, providing care for orthopedic trauma patients on two surgical orthopedic wards and two orthopedic rehabilitation wards. Inclusion criteria encompassed patients who were aged 60 years or older, medically stable, capable of sitting out of bed within 72 h of admission, and eligible for mobilization by nursing staff based on physiotherapy assessment. Moreover, prior to admission, individuals needed to be mobile, defined as being able to walk across a medium-sized room (approximately 3–4 m) with or without use of a walking aid. Furthermore, written informed consent was necessary for participation, which meant participants did not have significant cognitive impairment or delirium, as measured by the 4-AT test or documented in their medical notes. Study exclusion criteria were: individuals who were unable to mobilize with assistance prior to admission, those who could only be mobilized by a physiotherapist, who could not provide informed consent, and patients on an end-of-life or palliative care pathway.

### Data collection

Data collection included patient demographics and health profile such as frailty measured using the clinical frailty scale [21] (nine point scale from 1 = Fit to 9 = Severe frailty), sarcopenia, measured with the SARC-F tool [22] (five-item tool, maximum score of 10 where a higher score indicates worse physical function and a score ≥ 4 indicates likely sarcopenia), the modified Barthel index (measuring ten activities of daily living on a scale of 0-100, lower scores indicate higher levels of physical dependency) [23]. Data were collected at four time points: Time 1, baseline defined as two weeks prior to admission/injury, Time 2 on recruitment to the study, Time 3 at hospital discharge and Time 4 at 6–8 weeks following hospital discharge. The main outcome of interest was return to pre-injury functional capability [11].

Accelerometer data was collected on participants over 2–4 consecutive days on recruitment to the study using the StepWatch 4 which is validated for low gait speed [17].

Baseline data were collected on participating patients on the four wards over 6–8 weeks (control group), the FCB as outlined above was implemented on each ward over 6–8 weeks to embed the changes into ward routines, post intervention participants were recruited (experimental group) on these wards over 6–8 weeks. Baseline data began in November 2019 and final data collection was completed by November 2020.

### Feature extraction

**Fig. 1:**
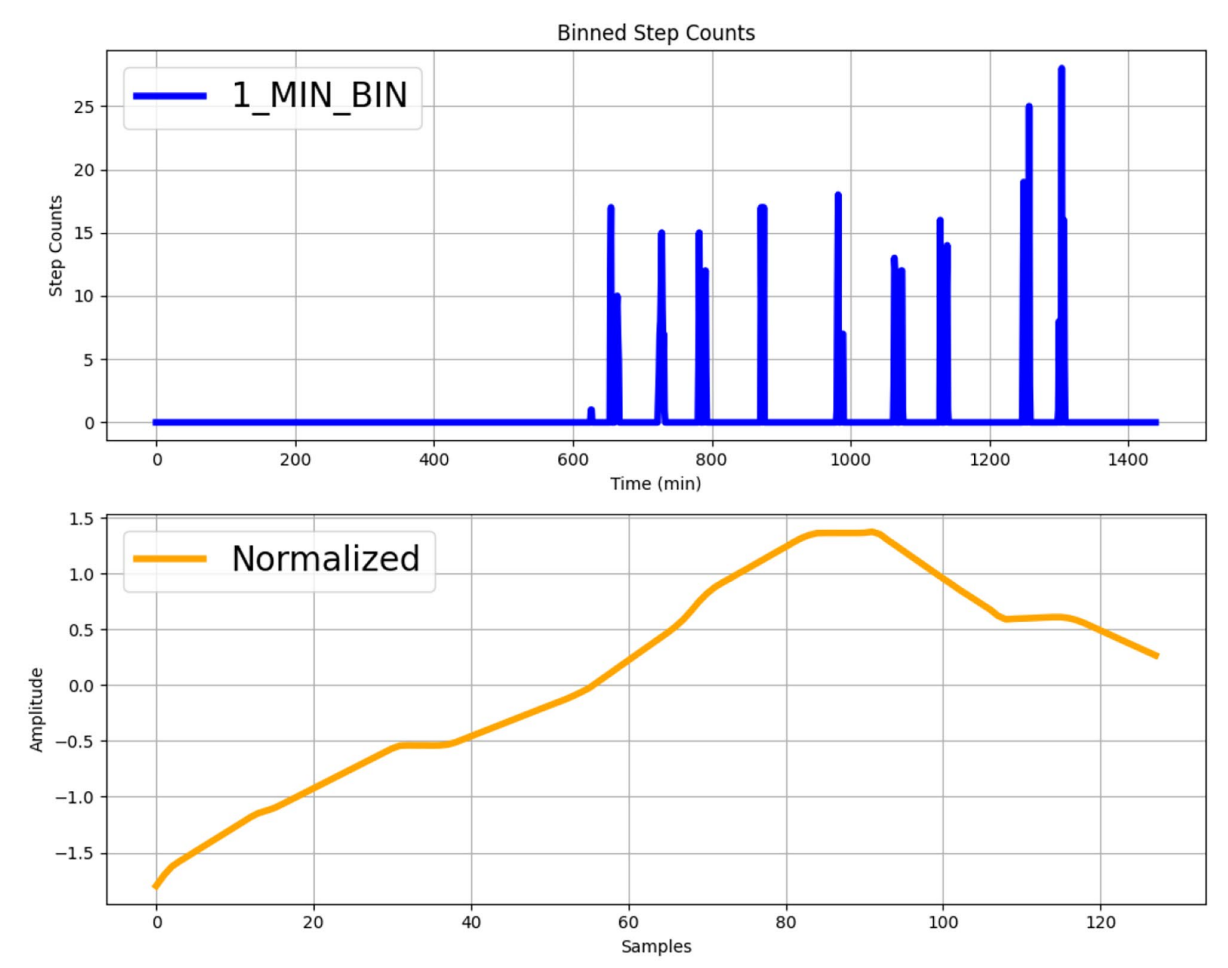
Example of step counts data collected over a day from a patient (above), and preprocessing and normalization of step count data (below)

Each patient’s accelerometer data consisted of a comma separated values (CSV) file containing daily aggregated gait variables (Table 1, parameters #1-#17), depending on how many days the patient wore the StepWatch 4. Additionally, the binned step counts for each day were provided in intervals of 10 s and one minute (Fig. 1, above). Statistical features (Table 1) were extracted from the data of each participant. Python 3.8.10 was used for this analysis along with the Numpy (1.23.1) and Nolds (0.5.2) packages [24]. The step counts binned in one-minute-long intervals for each day were standardized, averaged using a sliding window with a length of 50 samples, and down-sampled to a fixed length of 128 data points (Fig. 1, below). Non-linear measures for dynamical systems (Table 1, #18-#26) including entropy measures were calculated from the resulting one-dimensional time series to capture temporal dynamics and patterns in the data sequences. Non-linear dynamics measures have been widely adopted in human movement and gait analysis in order to quantify stability or complexity of movement and have been especially adopted with accelerometer systems [25–27]. For instance, Lyapunov exponents have a long history of being used as an assessment of walking stability and personal locomotive ability when applied on accelerometry [28]. In this analysis, the Lyapunov exponents estimated through the algorithm of Rosenstein et al. [29] and Eckmann et al. [30] were used. Likewise, entropy measures (such as sample entropy) measure the complexity of a time-series and offer an alternative method for appraising side-to-side dissimilarities in limb activity between sides [31]. Often employed to assess physiological parameters, entropy analysis has additionally been integrated into gait assessment across a range of medical conditions [31]. The Hurst exponent is a measure of the “long-term memory” of a time series and it can be used to determine the measure by which the time series deviates from a random time series. Its application to human gait for observation of aging and neurodegenerative diseases was shown in [32] and [24] was adopted for its implementation. Instead, the Detrended Fluctuation Analysis (DFA) [24] measures the Hurst parameter *H*, which is very similar to the Hurst exponent. The main difference is that DFA can be used for non-stationary processes (e.g., mean and/or variance change over time). Finally, the Grassberger-Procaccia algorithm [33] was used for estimating the correlation dimension, which is a fractal dimension, and it is characteristic of the set of points [34]. The mean, median, maximum, minimum, standard deviation, and range of the features and daily variables were calculated for each subject. The only exception was the stride length variable, where these statistics were not computed since it had a repeated value, resulting in identical values for all these features. A complete list of features collected for each participant is given in Table 1, which were then utilized in a binary classification ML model to determine whether the data originated from a subject in the pre- or post- intervention group.

### Data analysis

The data was randomly split into training and test sets in the ratio of 80%/20% such that no data from subjects in the training set would appear in the test. Additionally, stratified group k-fold cross-validation with k = 5 was carried out on the training set using balanced accuracy as the performance metric. The model pipeline included feature processing and selection by standardization and according to the highest ANOVA F-value scores, respectively. The number of selected features was set to 4 to prevent overfitting. The partitioning of data into training and test and subsequent model training and evaluation phase was repeated for 100 iterations to obtain a more robust validation of the model that is agnostic to the random nature of the split and ensure an accurate assessment of its generalizability. The number of iterations was chosen by experimental observation as the average accuracy was seen to converge. This process was performed for both a logistic regression classifier with an L2 penalty term and a Random Forest (RF) binary classifier. For the Random Forest, the number of trees, the maximum tree depth, the minimum number of samples required to split an internal node, and the minimum number of samples required to be at a leaf node, were set to 100, 2, 20, and 10, respectively.

Furthermore, SHAP (SHapley Additive exPlanations) analysis [35, 36], a concept from cooperative game theory that has been adapted for use in machine learning to provide a unified way of explaining the predictions made by complex models, was performed. An arbitrarily selected iteration of each model was chosen for this analysis to show the contribution of individual features in the models’ predictions and to gain a better interpretability and understanding of the models. SHAP values, thus, provided a framework for attributing the contribution of each feature in a prediction to the final model output.

### Statistical analysis

Categorical variables are described by their counts and percentages in each category, while continuous variables are presented using their medians and interquartile ranges (IQR). Pre versus post differences in patient demographic and health profile are obtained for each category and reported alongside p-values. A significance level of 0.05 was used for all comparisons. All analyses were conducted using IBM SPSS Statistics 24.

**Table 1:** Variables from StepWatch 4 included in daily aggregated data CSV file (#1 - #17) and features calculated from 1 min bin CSV file (#18 - #26) (the mean, median, maximum, minimum, standard deviation, and range was computed for each)

#	Parameter	Description
1	Cadence (CADENCE)	Steps per minute
2	Steps per day (STEPS_DAY)	Total number of steps taken on the leg wearing the StepWatch
3	Minutes active (MINUTES_ACTIVE)	Minutes of the day with at least one step
4	Percent time inactive (PERCENT_TIME_INACTIVE)	Percent of 24-hour day with no walking
5	Percent time in low activity (PERCENT_TIME_IN_LOW_ACTIVITY)	Percent of 24-hour day walking at a low activity cadence (default is 1–15 steps / minute)
6	Percent time in medium activity (PERCENT_TIME_IN_MED_ACTIVITY)	Percent of 24-hour day walking at a medium activity cadence (default is 16–40 steps / minute)
7	Percent time in high activity (PERCENT_TIME_IN_HIGH_ACTIVITY)	Percent of 24-hour day walking at a high activity cadence (default is 41 or greater steps / minute)
8	Stride velocity peak (STRIDE_VELOCITY_PEAK)	Peak velocity based on the minute with the most steps
9	Cadence average (CADENCE_AVERAGE)	Average cadence based on all minutes with at least 1 step
10	Cadence median (CADENCE_MEDIAN)	Median cadence based on all minutes with at least 1 step
11	Stride length (STRIDE_LENGTH)	Estimated in clinic through device calibration procedure
12	Max 60 (MAX_60)	Average cadence of the most intensive continuous 60 min of the day
13	Max 20 (MAX_20)	Average cadence of the most intensive continuous 20 min of the day
14	Max 5 (MAX_5)	Average cadence of the most intensive continuous 5 min of the day
15	Max 1 (MAX_1)	Highest cadence achieved in the day
16	Peak performance index (PEAK_PERF_INDEX)	Average cadence of the most intensive 30 individual minutes in the day
17	Gait speed (GAIT_SPEED)	Highest speed (in $\:km/hr$) achieved in the day calculated from $\:Max\:1\times\:Stride\:length$
18	Lyapunov exponent [29]	Estimate of the largest Lyapunov exponent (LYAP_E)
19	Lyapunov exponent 1 [30]	Lyapunov exponents (LYAP_E1, LYAP_E2, LYAP_E3, LYAP_E4)
20	Lyapunov exponent 2 [30]	
21	Lyapunov exponent 3 [30]	
22	Lyapunov exponent 4 [30]	
23	Sample entropy (SAMPLE_ENTROPY) [24]	Sample entropy of the data
24	Hurst exponent (HURST_RS) [24]	Estimated Hurst exponent using a rescaled range approach
25	Correlation Dimension (CORR_DIM) [33, 34]	Correlation dimension estimated as the slope of the line fitted to log(r) vs. log(C(r)) where the correlation sum C(r) is the fraction of point pairs in the phase space with a distance smaller than each specified radius r and the default for r is a list of values created by successively multiplying a minimum value ($\:0.1\times\:\text{s}\text{t}\text{d})$ by a factor of 1.03 until a maximum value ($\:0.5\times\:\text{s}\text{t}\text{d})$ is reached
26	Detrended fluctuation analysis (DFA) [24]	Estimate alpha for the Hurst parameter

## Results

### Patient characteristics

In the original study, 120 participants were recruited as part of the surgical population (59 pre-intervention and 61 post-intervention) [11]. The dataset analyzed in this study were 113 participants for whom complete accelerometry data were available (57 pre-intervention and 56 post-intervention). Days with less than 24 h of wear-time and participants with less than 1 full day of data were excluded from the analysis. Characteristics of the patients included in this sub-group analysis are shown in Table A1; there were no significant differences between this sub-group of 113 participants and those in the larger study [11]. The median age was 78 years, the majority were female and hip fracture was the most frequent injury sustained in the pre and post intervention groups. Participants in the post intervention group tended to have more long term conditions and a corresponding higher level of polypharmacy. At baseline there were similar scores on the SARC-F, but the clinical frailty scale indicated the post intervention group were slightly more frail. On enrolment to the study, the mBI indicated the baseline group had slightly higher dependency level than the post intervention group, while at 6–8 weeks follow-up post hospital discharge the mBI had improved in both groups.

There were no significant differences between the groups in other characteristics, the post intervention group tended to have a longer hospital stay (25.5 days) versus the pre-intervention 21.5 days. Multiple factors, other than readiness for discharge, influence hospital stay, including the impact of COVID-19 on community services required to support home discharge.

### Machine learning model

**Fig. 2:**
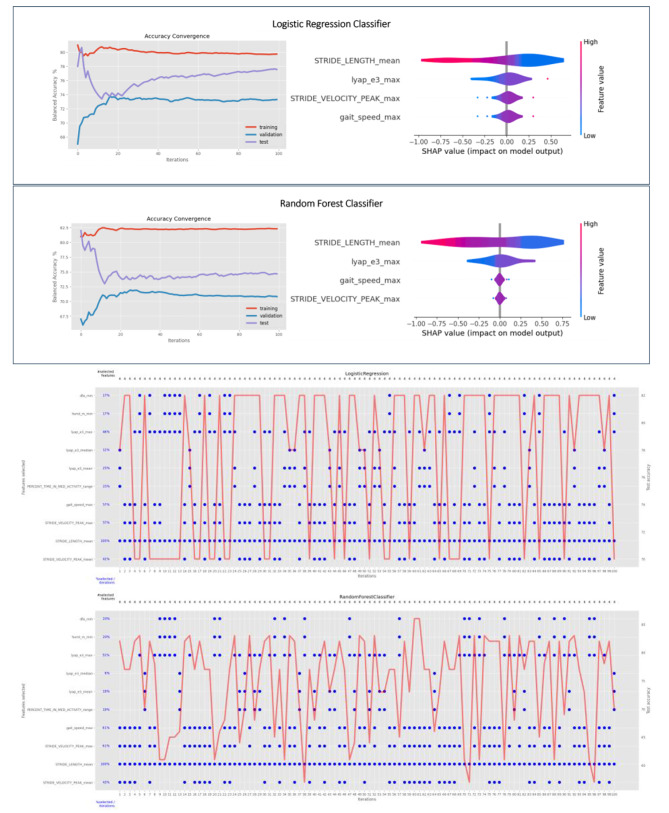
Top) Convergence of average accuracy over 100 iterations of random training/test splits (left) and SHAP analysis for one randomly selected iteration (right). Bottom) Balanced accuracy and features selected for each iteration. Top: Logistic regression, Bottom: Random Forest

The average balanced accuracy achieved using a Random Forest classifier was 82.3 ± 1.7% for the training and 74.7 ± 8.2% for the test set with no overlap in subjects between the two sets. Similarly, the average balanced accuracy achieved using a logistic regression classifier was 79.7 ± 1.9% (on the training set) and 77.6 ± 5.5% (on the final test set). The proximity between the results obtained for the training and test sets of the logistic regression model shows a lack of overfitting. However, the larger gap for the Random Forest model indicates more overfitting and a worse performance. The convergence of the model performance across the 100 repetitions is shown in Fig. 2 highlighting the overall robustness of the models. SHAP values obtained for one randomly selected iteration are also shown in Fig. 2 for both classifiers. The range of SHAP values for the four most commonly selected features chosen by both models, e.g., the STRIDE_LENGTH and the maximum of STRIDE_VELOCITY_PEAK, GAIT_SPEED, and LYAPUNOV_EXPONENT 3 (LYAP_E3), are described in Table 2.

**Table 2:** Range of SHAP values for the four most commonly selected features chosen by the random forest and logistic regression models

Model	Feature Name	Range (SHAP Values)
Random Forest	STRIDE_LENGTH_mean	-0.7 to 0.52
	STRIDE_VELOCITY_PEAK_max	-0.08 to 0.08
	GAIT_SPEED_max	-0.1 to 0.11
	LYAP_E3_max	-0.36 to 0.39
Logistic Regression	STRIDE_LENGTH	-0.74 to 0.41
	STRIDE_VELOCITY_PEAK_max	-0.33 to 0.3
	GAIT_SPEED_max	-0.33 to 0.3
	LYAP_E3_max	-0.4 to 0.46

**Table 3:** Features chosen by logistic regression and Random Forest models and frequency of selection (% of iterations)

Feature Name	Logistic Regression (% of iterations selected)	Random Forest (% of iterations selected)
STRIDE_VELOCITY_PEAK_mean	42%	36%
STRIDE_LENGTH_mean	100%	100%
STRIDE_VELOCITY_PEAK_max	57%	60%
GAIT_SPEED_max	57%	60%
PERCENT_TIME_IN_MED_ACTIVITY_range	25%	26%
LYAP_E3_mean	25%	26%
LYAP_E3_median	12%	16%
LYAP_E3_max	46%	48%
HURST_RS_min	17%	14%
DFA_min	17%	14%

For both models, the value of the variable STRIDE_LENGTH was chosen in 100% of all iterations. Both models also selected the maximum and mean of the variables STRIDE_VELOCITY_PEAK, GAIT_SPEED and LYAP_E3 with a frequency as shown in Table 3. The range of PERCENT_TIME_IN_MED_ACTIVITY, the median of LYAP_E3, and the minimum of HURST_EXPONENT (HURST_RS) and DETRENDED_FLUCTUATION_ANALYSIS (DFA) were also selected, albeit less frequently. A visualization of the features selected across the 100 iterations is shown in Fig. 2 (note that features never selected are not included in the visualization).

## Discussion

This paper explored the feasibility of using machine learning approaches to objectively differentiate the mobilization patterns, measured via accelerometer sensors, of patients pre- and post-the frailty care bundle intervention. The Random Forest classifier demonstrated an average balanced accuracy of 82.3% (± 1.7%) during training and 74.7% (± 8.2%) for the test set. In comparison, the logistic regression classifier achieved a training accuracy of 79.7% (± 1.9%) and a test accuracy of 77.6% (± 5.5%). The logistic regression model demonstrated less overfitting compared to the Random Forest model and better performance on the hold-out test set. Stride length was consistently chosen as a key feature in all iterations for both models, along with features related to stride velocity, gait speed, and Lyapunov exponent, indicating their significance in the classification. Overall, the performance was deemed satisfactory since the best performing classifier was able to distinguish between patients pre- and post-intervention with greater than 75% accuracy. This novel approach to analyzing patient mobilization data can better illustrate the effect of early mobilization strategies and improve prediction of functional recovery or risk of HAD.

Interventions aimed at reducing HAD among older adults in acute care settings have shown promising results, including a reduction in the risk of declining activities of daily living (ADL), nursing home residence, and mortality rates. While these outcomes are based on low-quality evidence, as summarized by two systematic reviews [8, 37], they hint at potential longer-term benefits of such programs, albeit with some uncertainties. Similarly, geriatric care models such as comprehensive geriatric assessment (CGA), aim to provide a tailored and person-centered approach to care that combines attention on fundamental care with medical and pharmacological management to deliver coordinated and integrated care plans. Yet, recent analysis indicates variable and short-term impacts on quality of life, carer burden, length of hospital stay, and remaining at home [38]. A problem with these types of multi-component interventions is it can be a ‘black box’ of what works and there is insufficient measurement of intervention fidelity (i.e., to what extent were the different components of the intervention delivered as intended) [39].

Multiple component interventions such as the FCB or CGA, require further analysis to understand which intervention components contribute most to the observed outcomes. The FCB took a theory-based approach to address specific challenges in caring for older patients during hospitalization. These challenges primarily involve the consistent prioritization of fundamental care aspects like mobility, nutrition, and cognition tailored for each ward and team to ensure implementation over competing demands on nursing resources. Remarkably, this study stands as one of the few interventions aiming to simultaneously enhance all three key elements of HAD [8]. A recent randomized trial, the CHERISH study (Collaboration for Hospitalized Elders Reducing the Impact of Stays in Hospital) [40], similarly targeted mobilization, nutrition and cognition. The results demonstrated a reduction in incident delirium but no impact on length of hospital stay or other adverse events. One limitation of the CHERISH study is that there was no patient level data collected on mobilization, thus there was no indication if mobilization strategies were effectively implemented at the patient level [40]. In summary, these findings in past literature underscore the importance of tailored interventions and multidisciplinary approaches in mitigating HAD among older adults in acute care settings, but there is a need to focus on intervention fidelity.

From a clinical and research perspective, we need better and real-time data on early indicators of HAD such as reduced mobilization. The ability to accurately measure and more efficiently analyze mobilization data is essential to provide clinical staff (nurses, physiotherapists, and doctors) with usable information to tailor patient care plans and intensity of physiotherapy and nursing interventions. The machine learning model presented in this paper can help push this field of research forward. Despite the large number of publications adopting wearable devices in pre- and post-operative settings [12–17, 41] very limited research has been carried out on using machine learning on the data collected in those settings from wearable devices. For instance, only Halfwerk et al. [12] adopted machine learning to classifying various patient activities (e.g., lying in bed, sitting in a chair, standing, walking, cycling on an exercise bike, and walking the stairs) to quantify in-hospital patient mobilization after cardiac surgery using accelerometers. Thus, this study highlights further opportunities beyond simple huma activity recognition in the adoption of machine learning into this field.

The results of the present analysis indicate that a logistic regression classifier achieved reasonably high accuracy in predicting the outcomes. In the context of this study, ‘reasonably high’ describes the accuracy exceeding 75% where 50% would be expected by random chance. On the test set, the average accuracy was comparable to the one obtained on the training set, indicating a lack of overfitting; moreover, there was no overlap in subjects between the training and test sets, ensuring a fair evaluation of the model’s potential performance on unseen data. The Random Forest model showed higher overfitting due to the limited amount of patients’ data collected in the study and the data-hungry nature of the model due to its non-linearity.

In terms of feature importance, the study found that certain features consistently played a crucial role in both classifiers. Specifically, the stride length was always selected for both classifiers. This suggests that this feature has a significant impact on the model’s predictions. Additionally, the maximum peak velocity based on the minute with the most steps and the highest gait speed achieved in the day were consistently selected in both models, indicating significant importance in the decision-making process. On the other hand, certain features were selected less frequently, making them less influential in the models’ predictions. Features based on the entropy and other non-linear dynamic measures of the binned step counts, and statistical features based on step counts were selected less often, indicating that patterns directly relating to these measurements were not as relevant as initially hypothesized. This may be due to the fact that step counts could not provide an overall picture of the walking capabilities of the patients and, therefore, raw accelerometry data may be more useful for those non-linear dynamic measures. We propose that numerous other factors can impact walking activity in hospital settings, which is supported by the findings of McCullagh et al. in their observational study on predictors of a shorter length of stay among older medical inpatients [42]. Notably, their research revealed a greater variability in step-count within individual patients than across the entire cohort of 154 subjects. Our findings further suggest that the average daily step count in and of itself is not a reliable indicator of recovery. However, it is worth pointing out as well the difference between cohorts investigated in [42] (medical patients) and this study (surgical population); indeed, gait may be impacted by recovery from surgery, with pain and ability to weight bear being significant factors on mobility compared to medical patients who have not experienced an acute traumatic injury — a nuance that requires careful interpretation by nursing and geriatric care teams.

It is important to highlight that stride length is not a metric calculated by the adopted device but was inputted by the clinical operators before attaching the device on the patients based on a calibration process similar to the one adopted in [43] where the average stride length was estimated by measuring the travelled distance after 10 steps and dividing it by 10. However, there are many studies in literature which adopted ankle-worn accelerometers to estimate stride length even in geriatric populations [44, 45], thus the automatic estimation of this metric may be integrated in future ad-hoc wearable solutions.

Based on the SHAP analysis, the intervention effect was evident in both models. The model interpreted a higher maximum peak velocity and gait speed, and a lower stride length, to be significant indicators of a subject post-intervention. There is insufficient information here to state the implication of a decreased stride length. A shorter stride length has commonly been identified as a significant predictor of life-threatening clinical events in older adults [46], however, it could be associated with rehabilitation or recovery in this surgical patient population. A shorter stride length may be due to balance concerns by the patients or possibly by pain (for example due to tightening of the tendons across hip); however, another randomized controlled trial on patients with hip fracture has not shown a similar reduction in stride length following surgery [47]. Future research should explore the effects on stride length post hip fracture considering that researchers have mostly focused on gait speed [48].

The results suggest that the intervention had an effect on the participants’ gait speed and stride length. This was reflected in the predictive models, where certain features consistently contributed to the model’s decisions, indicating their importance in distinguishing between the two groups. The present study successfully developed machine learning models for the objective classification of mobilization patterns, as obtained via accelerometer sensors, between patients in pre- and post-intervention. The study provides valuable insights into the factors that influenced the effectiveness of the HAD intervention, which can inform future refinement of the frailty care bundle tested in this study. Outside of the current study, there are important clinical implications for nursing and geriatric care. As mentioned previously, the current approaches to measuring patient mobilization in clinical settings are inadequate, with the incidents of falls or pressure ulcers most frequently reported as surrogate markers of mobility [49]. The machine learning approach described in this paper offers researchers a means of more efficient and accurate measurement of mobilization in health care settings and supports the development of more useful real-time measure of patient progress and preventive risk reduction strategies than waiting for the occurrence of adverse events, such as falls or pressure ulcers.

By providing more efficient and accurate measures of patient mobilization, these models can support real-time assessment of patient progress and facilitate the implementation of preventive risk reduction strategies. Such advancements in mobility assessment hold significant potential to enhance patient care and improve outcomes in acute care settings, underscoring the importance of interdisciplinary collaboration between nurses, geriatricians, physiotherapist and the wider MDT.

It is important to stress some limitations in the data. Even though the dataset consisted of 113 subjects, the sample size was still relatively small and further analysis with larger data collections across different countries are required to validate the generalizability of the observed insights. Moreover, even though the accelerometer adopted in this study was validated in several publications [17], the device could only provide summary statistics of step counts in bins of 10 s and 1 min instead of the raw accelerometry time-series signal. It can be assumed that those time-series signals should contain further information which, if analyzed with sufficient computational power, could provide further meaningful insights into the problem of objectively differentiating the mobilization patterns via machine learning and accelerometer sensors.

## Conclusions

This study highlights the vulnerability of older adults in acute care settings, where extended hospital stays and associated increased risk of functional decline are prevalent. The FCB intervention, which emphasizes mobility, in addition to nutrition, and cognition, indicated promising results in preserving functional capability. The results demonstrate the accuracy of predictive models in objectively differentiating the mobilization patterns, measured via accelerometer sensors, of patients pre- and post-intervention, with specific features consistently playing a crucial role in decision-making. Importantly, the intervention was associated with higher gait speed and reduced stride length. However, the question of whether these changes signify an adaptive process leading to long-term improvements in outcomes remains unanswered. The machine learning models developed in this study offer valuable insights for future interventions, contributing to improved health outcomes for this at-risk patient group.

## Electronic supplementary material

Below is the link to the electronic supplementary material.

Supplementary Material 1

## Data Availability

The anonymised data are available in Zenodo at [50].
