# Supplementary material for: Treatment effect analysis of the Frailty Care Bundle (FCB) in a cohort of patients in acute care settings
Source: Aging Clin Exp Res. 2024 Sep 10;36(1):187. doi: 10.1007/s40520-024-02840-5 (PMC11387438; doi:10.1007/s40520-024-02840-5)
Supplement: Supplementary file 1 — Supplementary Material 1 [file 40520_2024_2840_MOESM1_ESM.docx]

**Table A.1:** Patient demographic and health profile

| **Characteristic** | **Pre (n=57)** | **Post (n=56)** | **p-value** |
| --- | --- | --- | --- |
| ***Age (years), median (IQR)*** | 79 (73, 85) | 78 (73, 83) | 0.7 |
| ***Sex*** |  |  | 0.116 |
| Female, % | 77.2% | 63.6% |  |
| Male, % | 22.8% | 36.4% |  |
| ***BMI (kg/m2), median (IQR)*** | 24.84 (21.43, 29.3) | 26.09 (24.41, 30.6) | 0.19 |
| ***Living situation*** |  |  | 0.543 |
| Lives alone | 38.6% | 45.5% |  |
| Lives with other family | 15.8% | 10.9% |  |
| Lives with spouse/ partner | 45.6% | 40% |  |
| Lives other | - | 3.6% |  |
| ***Reason for admission*** |  |  | 0.301 |
| Hip fracture / repair | 64.9% | 50.9% |  |
| Lower limb fracture / injury | 21.1% | 20% |  |
| Spinal fracture | 3.5% | 1.8% |  |
| Upper limb fracture | 1.8% | 1.8% |  |
| Wound infection / Soft tissue injury | 7% | 16.4% |  |
| Other | 1.7% | 9.1% |  |
| ***Early-warning score, median (IQR)*** | 0 (0, 1) | 0 (0, 0) | 0.18 |
| ***SARC-F score, median (IQR)*** | 2 (1, 4) | 2 (1, 5) | 0.19 |
| ***Clinical Frailty Score prior to admission, median (IQR)*** | 3 (2, 4) | 4 (3, 5) | 0.238 |
| **Modified Barthel Index 2 weeks prior to admission*, median (IQR)*** | 95 (85, 100) | 100 (95, 100) | 0.18 |
| **Modified Barthel Index on enrolment*, median (IQR)*** | 60 (48.75, 65) | 65 (50, 70) | 0.318 |
| **Modified Barthel Index 2 weeks at discharge or 6-8 weeks follow-up*, median (IQR)*** | 85 (67.5, 92.5) | 85 (75, 95) | 0.413 |
| ***Total days in hospital, median (IQR)*** | 21.50 (14.75, 28.00) | 25.50 (14.50, 33.00) | 0.257 |
| ***Average grip strength (kg), median (IQR)*** | 19 (13.58, 23.00) | 18.34 (14.24, 22.16) | 0.92 |
| ***Average grip strength at discharge (kg), median (IQR)*** | 20 (14.67, 24.00) | 17.60 (13.80, 23.75) | 0.4 |
| ***Gait time 4 metre walk at recruitment (s), median (IQR)*** | 25.41 (14.87, 36.89) | 19.78 (15.55, 27.78) | 0.51 |
| ***Gait speed 4 metre walk at recruitment (m/s), median (IQR)*** | 0.16 (0.11, 0.27) | 0.20 (0.14, 0.26) | 0.285 |
| ***Gait time 4 metre walk at discharge (s), median (IQR)*** | 16.07 (8.90, 22.48) | 14.20 (10.30, 19.63) | 0.26 |
| ***Gait speed 4 metre walk at discharge (m/s), median (IQR)*** | 0.25 (0.18, 0.45) | 0.29 (0.20, 0.39) | 0.62 |
| ***Polypharmacy (>5 medication)*** | 64.9% | 90.9% | 0.003 |
| ***Number medications per day, median (IQR)*** | 5 (3, 7) | 7 (5, 10) | 0.001 |
| ***Number co-morbidities, median (IQR)*** | 4 (3, 6) | 4 (3, 4.25) | 0.378 |
| ***Hypertension*** | 61.4% | 70.9% | 0.028 |
| ***Angina*** | 1.8% | 1.8% | 0.116 |
| ***Heart attack (MI)*** | 0% | 5.5% | 0.021 |
| ***Diabetes on treatment*** | 17.5% | 16.4% | 0.117 |
| ***Stroke and transient ischemic attack*** | 8.8% | 18.2% | 0.031 |
| ***Hemiplegia*** | 3.5% | 1.8% | 0.059 |
| ***Neurological condition (Parkinson’s, MS)*** | 7.0% | 7.3% | 0.115 |
| ***Irregular heart rhythm*** | 22.8% | 20.0% | 0.115 |
| ***Other CVD (e.g., heart failure, peripheral vascular disease)*** | 24.6% | 41.8% | 0.034 |
| ***Cataracts*** | 21.1% | 12.7% | 0.138 |
| ***Glaucoma and age-related macular degeneration*** | 5.3% | 3.6% | 0.11 |
| ***Arthritis*** | 33.3% | 45.5% | 0.03 |
| ***Osteoporosis*** | 57.9% | 63.6% | 0.06 |
| ***Cancer*** | 22.8% | 12.7% | 0.056 |
| ***Varicose leg ulcer*** | 3.5% | 5.5% | 0.098 |
| ***Respiratory condition (COPD, asthma)*** | 19.3% | 20.0% | 0.112 |
| ***Mental health: Depression / anxiety (received treatment)*** | 15.8% | 9.1% | 0.076 |
| ***Smoker (current)*** | 8.8% | 7.3% | 0.115 |
| ***Other Diseases*** |  |  |  |
| Renal disease | 5.3% | 3.6% | 0.563 |
| Liver disease | 0% | 0% | 0.31 |
| GI peptic ulcer | 14.0% | 9.1% | 0.467 |
| Haematology / anticoagulants (e.g., warfarin) | 10.5% | 14.5% | 0.442 |
| Other | 12.3% | 18.2% | 0.354 |

Key: IQR, Interquartile range; BMI, Body Mass Index; MI, Myocardial infarction; MS, Multiple Sclerosis; CVD, Cardiovascular disease; COPD, Chronic Obstructive Pulmonary Disease; GI, Gastro-intestinal;
